# Evaluating the feasibility and acceptability of the Informed Health Choices-Cancer programme: A pilot randomised trial protocol

**DOI:** 10.1371/journal.pone.0333871

**Published:** 2025-10-09

**Authors:** Mengqi Li, Marie Tierney, Claire Beecher, Maura Dowling, Austin G. Duffy, Caitriona Duggan, David Robert Grimes, Avril Kennan, Claire Kilty, Allen Nsangi, Andrew D. Oxman, Derek C. Stewart, Elaine Toomey, Declan Devane

**Affiliations:** 1 Centre for Health Research Methods, School of Nursing & Midwifery, University of Galway, Galway, Ireland; 2 Health Research Board – Trials Methodology Research Network (HRB-TMRN), University of Galway, Galway, Ireland; 3 Evidence Synthesis Ireland and Cochrane Ireland, University of Galway, Galway, Ireland; 4 School of Nursing & Midwifery, University of Galway, Galway, Ireland; 5 Department of Medical Oncology, Mater Misericordiae University Hospital, Dublin, Ireland; 6 Department of Oncology, Portiuncula University Hospital, Galway, Ireland; 7 Biostatistics Unit, Discipline of Public Health and Primary Care, School of Medicine, Trinity College Dublin, Dublin, Ireland; 8 Health Research Charities Ireland (HRCI), Dublin, Ireland; 9 Irish Cancer Society, Dublin, Ireland; 10 Department of Medicine, College of Health Sciences, Makerere University, Kampala, Uganda; 11 Centre for Epidemic Interventions Research, Norwegian Institute of Public Health, Oslo, Norway; 12 College of Medicine, Nursing & Health Sciences, University of Galway, Galway, Ireland; Khyber Teaching Hospital, PAKISTAN

## Abstract

**Background:**

More than one-third of cancer-related health information is unreliable or misleading. With increasing health information seeking, the risks of misinformation exposure are growing. Educational approaches that are delivered clearly and in accessible formats are a promising way to strengthen critical thinking and decision-making skills, thereby helping to reduce the exposure of those impacted by cancer to misleading cancer information.

**Methods:**

This pilot randomised trial will assess the feasibility and acceptability of conducting a larger definitive trial evaluating the Informed Health Choices-Cancer (IHC-C) programme. The IHC-C is an online evidence-based education programme co-designed by stakeholders, including public and patient partners. It aims to equip people impacted by cancer (i.e., current patients, survivors, caregivers, and loved ones) with the skills and knowledge necessary to think critically about the reliability of health information and claims and make informed health choices. Participants will be randomised to either the IHC-C intervention group or a waitlist control group. The primary outcome of this pilot trial are feasibility (recruitment and retention rates, etc.) and acceptability (participant satisfaction and perceived usefulness, etc.). Demographic and cancer-related data will be collected to characterise the sample and inform recruitment strategies for a future definitive trial. Preliminary measures of critical thinking and decision-making skills will also be gathered to support the selection of key outcomes for the future trial.

**Discussion:**

This pilot trial will inform the design and conduct of a future definitive randomised trial. Insights gained will inform sample size estimations, refine recruitment strategies, optimise programme delivery, and improve data collection processes, ensuring a robust and scalable approach for the definitive trial.

## 1. Introduction

There were nearly 20 million new cases of cancer in 2022, and it is predicted that new cases of cancer will reach 35 million annually by 2050 [1]. People impacted by cancer have varying information needs throughout their cancer journey – from diagnosis through treatment to survivorship [2,3] – to best manage their health and make informed health decisions [4]. It is reported that one in three patients actively search for information themselves [5]. Among those impacted by breast cancer, more than 90% engage in information scanning through sources such as television, friends, and family, while over 60% seek information online or from healthcare providers [6].

Despite this significant information need, studies have identified cancer as one of the most concerning topics regarding the quality of health information [7–13]. The World Health Organization has labelled this an ‘infodemic’ of misinformation, defined as information that is inaccurate, outdated, incomplete, or misleading based on the best available evidence at the time, disseminated regardless of intent to mislead [14–16]. Notably, 52% of those impacted by cancer encounter conflicting information [17], and it is concerning that lower-quality and misleading content often garners more engagement than accurate information [12].

The widespread accessibility and rapid dissemination of health information makes it challenging to distinguish reliable from unreliable claims [18–20]. On an individual level, misplaced trust in unreliable claims can lead to delays in standard treatment and avoidable harm, including death. On a collective public level, misinformation about maintaining or improving health can and has altered attitudes, behaviours, and policies on crucial health issues, including cancer, leading to poorly informed choices.

Various strategies have been proposed to combat cancer misinformation, including collaboration with clinicians [21], direct participation of health professionals in social networks [22], and collaborative dialogue between healthcare providers, patients, researchers, and advocacy groups [23]. While health professionals play a crucial role in identifying misinformation, their efforts are limited by the sheer volume of misinformation. Consequently, the burden of identifying misinformation falls heavily on those impacted by cancer themselves [12].

While reducing the amount of information available may not be possible, education programmes have been suggested as an effective way to reduce the number and extent to which those impacted by cancer are exposed to or impacted by misinformation [14,16,24,25]**.** Low health literacy is associated with greater use of and trust in health information on social media [26]; therefore, such programmes seek to improve health literacy and enable better informed decision-making [6,27,28].

The Informed Health Choices-Cancer (IHC-C) programme [29–32] is an evidence-based online education initiative designed to equip individuals impacted by cancer with the skills and knowledge needed to critically assess the reliability of health claims and make informed decisions. The programme was systematically developed through a rigorous, multi-phase process that included stakeholder engagement, prioritisation of Key Concepts, co-design of content, and iterative pilot testing with refinement based on participant feedback. The programme is now ready for evaluation as an intervention in this randomised controlled trial (RCT).

This pilot RCT protocol details a trial to investigate the feasibility and acceptability of conducting a definitive trial of the effectiveness of the IHC-C programme. Data will be collected to inform the design and methodology of the definitive RCT.

## 2. Methods

### 2.1. Study design

**Fig 1:**
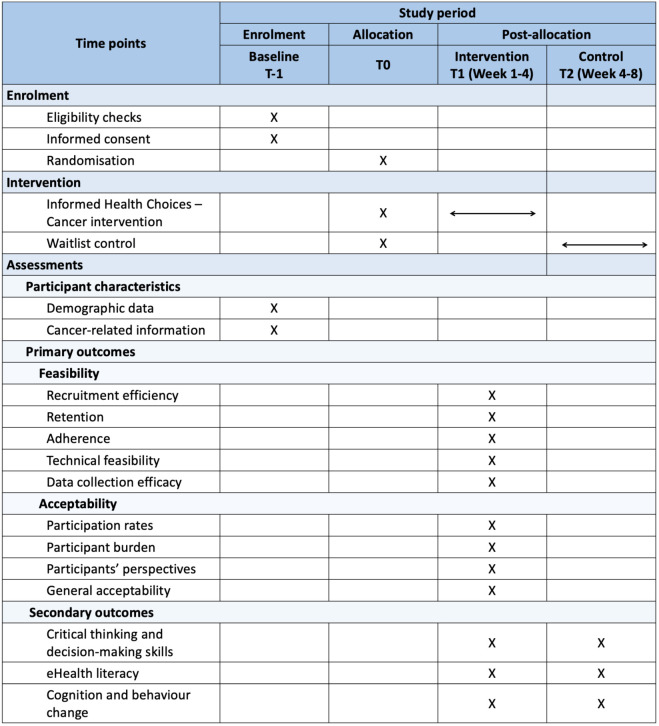
Schedule of enrolment, interventions, and assessments.

**Fig 2:**
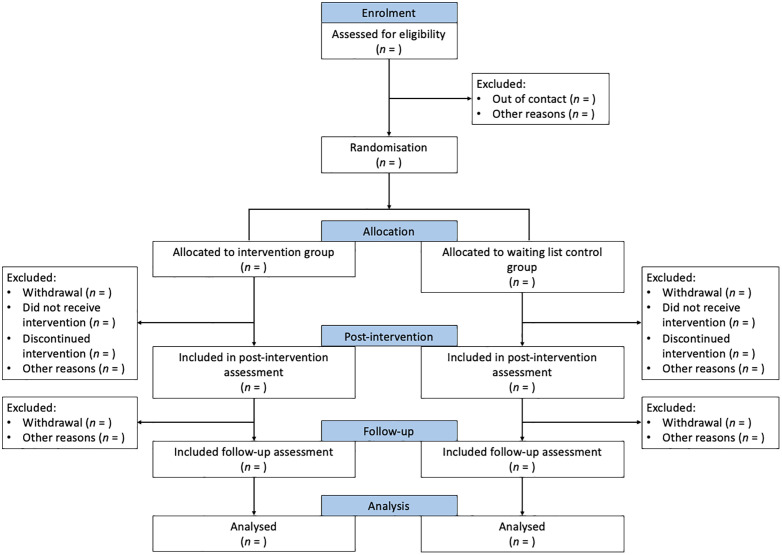
Flowchart.

This pilot randomised trial will investigate the feasibility and acceptability of conducting a definitive trial of the IHC-C programme. This protocol’s reporting follows the Standard Protocol Items: Recommendations for Interventional Trials (SPIRIT) guidelines (S1 File) [33]. The trial design adheres to the Consolidated Standards of Reporting Trials (CONSORT) statement and its adaptation for pilot trials and eHealth interventions [34–36]. Fig 1, developed in accordance with the SPIRIT reporting guidelines and adapted to the design of this pilot trial, presents the schedule of enrolment, interventions, and assessments. Fig 2 outlines the study flowchart, which will be presented in the publication of trial results.

### 2.2. Participants

Based on the previous stages of the IHC-C programme development, which involved stakeholder-led prioritisation and pilot testing with people impacted by cancer [31], we will recruit:

- Current patients of any type of cancer,
- Survivors of any type of cancer,
- Informal caregivers of a person with cancer,
- Loved ones of a person with cancer.

#### 2.2.1. Inclusion criteria.

- Age ≥ 18 years,
- Current patients and survivors – diagnosed with any type of cancer,
- Current patients – person currently undergoing treatment for any type of cancer,
- Survivors – those who have completed treatment and with or without current care/follow-up,
- Informal caregivers – those that provide the majority of unpaid, informal care, and self-identify as informal caregiver for a person diagnosed with cancer,
- Loved ones – family member, friend, or someone who cares about a person diagnosed with cancer, and who self-identifies as a loved one of a person diagnosed with cancer,
- Be able to commit to the study for at least four weeks,
- Can give informed consent,
- Can access the internet.

#### 2.2.2 Exclusion criteria.

- Currently involved in another similar study,
- Irregular and frequently changing caregivers (for informal caregivers).

### 2.3. Recruitment and obtaining informed consent

Recruitment is expected to take place between May and October 2025. Participants will be recruited through convenience sampling, utilising both online and offline methods. Online recruitment will involve targeted social media campaigns on suitable platforms, where posts will be designed to engage individuals impacted by cancer. The posts will direct potential participants to an online expression of interest portal. Offline efforts will include traditional media campaigns through press releases distributed to local and national media outlets. These releases will raise awareness about the trial within the broader community, particularly among those who may not be active online. Additionally, recruitment will be bolstered by outreach through cancer support centres, organisations, charities, and groups.

Interested individuals will be directed to an online survey where they can submit their email addresses. Participants who express interest will be emailed further information and an online consent form. Confidentiality and secure data handling practices will be clearly communicated at this stage.

### 2.4. Sample size

Following guidance for pilot trials [37–40], we will recruit 30 participants per group to allow for an anticipated 15%−25% attrition rate [32]. This target will provide sufficient numbers (20–25 participants per group) for assessing feasibility outcomes including recruitment, retention, and adherence rates [41–45].

### 2.5. Randomisation

After informed consent and baseline data collection, participants will be randomised to study arms using stratified randomisation based on cancer experience (current patients, survivors, informal caregivers, and loved ones) to ensure balanced representation across these groups. Participants will be randomly assigned in a 1:1 ratio to either the IHC-C intervention group or the waitlist control group through the QuestionPro survey platform [46]. To implement stratified randomisation, separate block paths will be configured in the platform’s survey logic for each stratum, with each stratum identified based on participants’ responses to a question about their cancer experience. Within each stratum-specific block, the platform’s automated block randomisation feature will be used, with the “evenly present blocks” setting enabled to maintain balance between groups to make sure the randomisation occurs independently within each stratum, consistent with standard stratified randomisation procedures, and reduces the risk of allocation imbalances that can occur in small-sample pilot trials. Allocation will remain concealed from researchers throughout the process.

Based on earlier recruitment for this programme, we anticipate a higher proportion of survivor respondents [32]. To avoid excluding these volunteers while still preventing extreme imbalance, we will not impose fixed numeric quotas per stratum. Instead, stratified randomisation will continue until the overall sample reaches N = 60. If any stratum remains under-represented (≤ 10 participants) when total enrolment nears 50, targeted advertising will be used to bolster that subgroup.

### 2.6. Blinding

Participants and study researchers will be blinded to group allocation at the time of recruitment and baseline assessments, as randomisation will occur only after these assessments are completed. However, for practical reasons, researchers will not remain blind to the allocation after randomisation, and participants will be informed of their group allocation – whether or not they are allocated to receive the intervention – immediately following randomisation. Due to the nature of the intervention, outcome assessors cannot be fully blinded, which will be acknowledged as a study limitation.

### 2.7. Intervention

#### 2.7.1. Informed Health Choices-Cancer programme.

The IHC-C programme was developed collaboratively with public and patient involvement (PPI) participants, oncologists, cancer nurses, cancer researchers, and educators. An ongoing iterative process of drafting, reviewing, revising, and refining ensured that the programme’s content was clear, comprehensible, and aligned with high educational standards while effectively communicating the intended learning outcomes. The programme underwent two rounds of pilot assessments with these key stakeholders before implementation in this trial.

**Fig 3:**
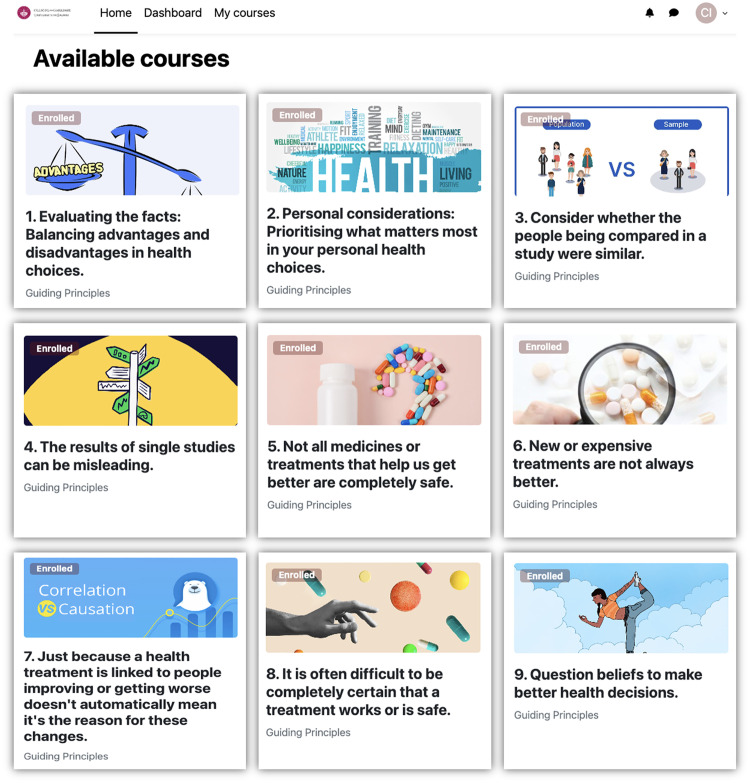
IHC-C programme on Moodle.

The IHC-C programme is a nine-unit online educational programme (see Fig 3) developed using a human-centred design (HCD) approach with iterative refinement cycles. It aims to help people impacted by cancer think critically about the reliability of health information and claims and make well-informed choices [32]. Hosted on the Moodle learning management system (LMS), the programme can be freely accessed from any location or device with internet access (see Fig 3).

**Fig 4:**
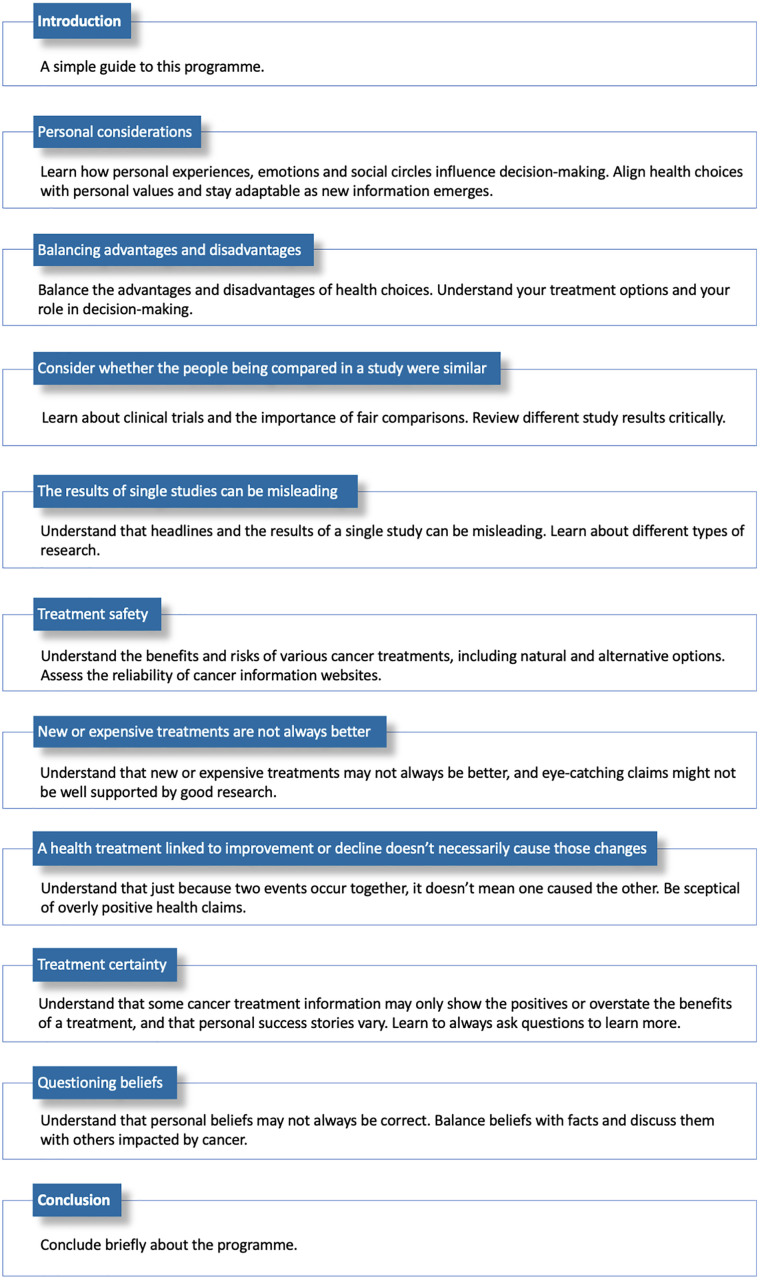
IHC-C programme units.

The nine units are derived from a suite of 49 Informed Health Choices (IHC) Key Concepts (KC), prioritised by the first stage of the IHC-C project for their relevance to those impacted by cancer [32]. The intervention has been designed based on evidence that incorporates various educational formats, such as narrated texts, videos, online articles, social media posts, and interactive activities, to provide comprehensive, relevant, cancer-specific information and knowledge. Each unit is on a specific topic and follows a consistent structure (see Fig 4), requiring 40–45 minutes of study time.

Participants can revisit each unit multiple times, with their progress and responses saved. Each unit concludes with a multiple-choice quiz related to the unit’s topic and learning outcomes. All participants are encouraged to complete the quiz, and correct answers with explanations are provided afterwards. Quiz results will be used to assess participants’ learning outcomes, while completion is encouraged, non-completion will not affect their participation in the programme. If participants experience difficulty completing the quiz (e.g., due to illness, literacy, or technical issues), they will be offered appropriate support or an alternative way to provide feedback.

#### 2.7.2. Intervention group.

Participants randomised to the intervention group will be given access to the IHC-C programme after allocation. The intervention will occur over four weeks. Participants will be encouraged to follow the intervention instructions and complete the programme at their own pace within the four weeks. Those who have not started or completed the programme will receive a follow-up email reminder at the beginning of the final week. Additional support will be offered to participants who show low engagement early in the intervention period, including automated reminders and, where appropriate, personalised follow-up emails to encourage ongoing participation. Email support will be available to all participants, with responses typically provided within 24 hours. A certificate of completion will be awarded to those who finish the programme.

#### 2.7.3. Waitlist control group.

Participants assigned to the waitlist control group will gain access to the IHC-C programme after four weeks, once the intervention group completes the programme and assessments. Participants in the waitlist group will then have four weeks to complete the programme, during which they will receive the same support, reminders, and resources as those in the intervention group. All data collection, including baseline and follow-up assessments, will be conducted using the same tools and methods to ensure comparability of outcomes across both groups. As the primary aim of this pilot study is to inform the design of a future definitive trial, this waitlist control design allows for a controlled comparison of feasibility and acceptability between groups during the intervention phase, while ensuring that all participants ultimately receive access to the programme.

### 2.8. Outcomes and measurements

#### 2.8.1. Participant characteristics.

Characteristics of participants will be collected through a self-report online survey developed by the research team (S2 File), which was also used in earlier stages of the IHC-C programme (31). The survey will gather the following information:

- Demographic data: age, gender, educational background, ethnic background, employment status, and internet accessibility.
- Cancer-related information: how the participant is impacted by cancer (as outlined in the ‘Eligibility criteria’ section), cancer diagnosis, time since diagnosis (for patients and survivors), and the relationship between current patients/survivors and their informal carers/loved ones.

#### 2.8.2. Primary outcomes.

The primary outcomes will assess feasibility and acceptability. Outcomes will be gathered through a structured survey, designed using standard recommendations for pilot RCTs and instruments from similar intervention studies, and tailored to this trial’s specific needs (S2 File).

Feasibility

- Recruitment efficiency: the number and proportion of participants who were approached, consented, deemed eligible, and randomised within the recruitment period. Data will be monitored using trial recruitment logs.
- Retention: the number and proportion of participants who remain in the programme from baseline to the final outcome assessment. Data will be collected from the online learning platform.
- Adherence: the extent to which participants follow the intervention protocol and engage with the programme, which includes login frequency, time spent on the platform, and interaction with programme materials. Data will be collected through the online learning platform, noting any missed or incomplete units and documenting the reasons for non-adherence via system-generated inactivity logs of the learning platform.
- Technical feasibility: the capability of the online platform to deliver the intervention and support user access. Data will be gathered through a participant survey evaluating ease of access and any technical problems encountered (S2 File). In addition, technical support logs will also record system issues and user-reported difficulties. We do not intend to extract backend data directly from Moodle for this pilot study.
- Data collection efficacy: the number and proportion of completed outcome assessments and identification of any issues with measurement tools. Data will be collected through the online learning platform.

Acceptability

- Participation rates: the number and proportion of participants who complete individual units and assigned activities. Data will be collected through the online learning platform.
- Participation burden: assessment of the effort required by participants to engage with the programme and complete tasks. Data will be gathered through participant survey questions regarding perceived efforts and whether participants found it acceptable. This will include both the burden of engaging with the intervention and completing outcome assessments.
- Participants’ perspectives: based on participant response to open-ended questions about participants’ experience with each unit and the overall programme. Data will be collected through survey questions.
- General acceptability: participants’ perceptions of the programme, including their views on its content, format, duration, overall acceptability, and willingness to recommend. Data will be collected through survey questions.

#### 2.8.3. Secondary outcomes.

This pilot trial will also collect outcomes intended for the future definitive trial, focusing on:

- Critical thinking and decision-making skills involve participants’ ability to appraise evidence, identify misleading claims, and assess the reliability of health information, enabling them to make well-informed decisions learned from the intervention. Data will be collected post-intervention using the structured evaluation tool *Critical Thinking in Cancer and Health Information Evaluation: Scenario-Based Assessment, which includes* four scenarios with 17 questions developed specifically for the IHC-C programme.
- eHealth literacy: participant’s experience using the internet for health information, measured with the 8-item eHealth Literacy Scale (eHEALS) [47] post-intervention.
- Cognition and behaviour change: participants’ self-reported changes in understanding and approach to engaging with information. Data will be collected through two specific questions.

#### 2.8.4. Assessment timeline.

Assessments will occur at four time points (see Fig 1):

- T − 1 (Baseline assessment): All participants will complete baseline assessments.
- T0 (Allocation): Randomisation and assignment to the intervention or waitlist control group. No assessments are conducted at this timepoint.
- T1 (Post-intervention: 4 weeks): Both groups complete assessments – intervention group after completing the programme, control group without programme access.
- T2 (Control access: 6 weeks): Waitlist control group receives programme access.

### 2.9. Statistical analysis plan

Data will be analysed following the intention-to-treat principle. Missing data will be addressed using multiple imputation techniques [48]. Data will be stored and processed using Microsoft Excel, SPSS 28.0, and NVivo 20 Pro. No serious negative outcomes are anticipated for participants; therefore, an interim analysis will not be conducted and no formal stopping rules have been established.

#### 2.9.1. Participant characteristics analysis.

Participants’ characteristics will be summarised using descriptive statistics. Means, standard deviations (SDs), medians, and interquartile ranges will be reported for continuous variables. Frequencies and percentages will be reported for categorical variables.

#### 2.9.2. Primary outcomes analysis.

Primary outcomes will be analysed using descriptively, using the same approach for descriptive statistics outlined above. Specifically,

- **Feasibility** outcomes will be analysed using counts and percentages, along with participants’ responses to survey questions. Recruitment efficiency will be deemed feasible if 80% of the recruitment target is met in terms of participants randomised. Retention will be considered successful if 70% of participants stay in the intervention. Adherence will be regarded as successful if 50% of participants spend at least 75% of the recommended time on the platform. Technical feasibility will be deemed successful if 80% of participants report no significant technical issues, defined as problems that prevent or severely hinder access or completion (e.g., frequent login failures or content not loading). Data collection efficacy will be considered successful if 90% of all required assessments are completed.
- **Acceptability** outcomes will be analysed using counts and percentages, along with participants’ responses to survey questions and a qualitative thematic analysis of open-ended responses. Participation rates will be deemed acceptable if they exceed 70% in each of the units and assigned activities. Participation burden will be considered acceptable if 75% of participants report that the effort required to engage with the programme is reasonable. Participants’ perspectives on the intervention will be assessed based on themes identified from their feedback on the open-ended survey questions. General acceptability will be regarded as successful if 75% of participants rate the programme as satisfactory or higher.

#### 2.9.3. Secondary outcomes analysis.

Critical thinking ability, decision-making skills and eHealth literacy will be analysed using survey scores, comparing mean changes at end of intervention between intervention and control group. Knowledge application and intended behaviours and self-efficacy will be assessed through survey responses.

#### 2.9.4. Subgroup and additional analyses.

Exploratory subgroup analyses may examine outcomes across the four participant categories (current patients, survivors, informal caregivers, and loved ones) using descriptive statistics.

### 2.10. Data management

All data for this trial will be collected online. Data management for this trial will adhere to the University of Galway’s Research Data Protection Policy [49]. Participants will be informed at the beginning of their participation in this trial about how their data will be handled. Data will be securely stored and accessible only by authorised personnel.

### 2.11. Ethical considerations

Potential participants will receive a participant information sheet outlining the study purpose, procedures, and time commitments. The sheet will detail participant expectations and rights, including withdrawal without consequence and potential benefits and risks. Contact information for the research team will be provided.

The sheet will explain that while personal identifiers are needed for study follow-up, all data will be accessible only to the research team, used solely for this study and related research, coded for analysis and reporting, and stored securely following institutional protocols.

Informed consent will be obtained through a web-based form after participants confirm they have read and understood this information.

Ethical approval for this pilot trial was obtained from the University of Galway Research Ethics Committee (Ref: 2024.12.018). This trial was registered with the ISRCTN Registry (Ref: ISRCTN17391470). Any substantial protocol modifications will be documented and reported when the pilot trial findings are published. Participants will be provided with a report of the trial outcomes and implications after the study.

## 3. Discussion

Using an online delivery to enhance accessibility [50], the IHC-C programme aims to help people impacted by cancer evaluate health information and make informed choices. This pilot study will assess the feasibility and acceptability of conducting a future definitive trial

Key limitations of this pilot study include potential selection bias due to online delivery, as participation is inherently limited to those with reliable internet access and appropriate digital literacy. Additionally, the waitlist control design does not control for attention or expectancy effects, which could influence participants’ outcomes independently of the intervention.

If feasible, findings will inform the design of a definitive trial support potential integration into routine cancer education and guide the development of similar interventions in other health contexts.

In conclusion, this pilot RCT will provide valuable insights into the feasibility and acceptability of the IHC-C programme as an intervention for those impacted by cancer. The results will inform refinements to the intervention and key methodological decisions for a future definitive trial, ultimately contributing to efforts to counteract cancer-related misinformation.

## Supporting information

S1 File Standard Protocol Items: Recommendations for Interventional Trials (SPIRIT) checklist. (PDF)

S2 File Informed Health Choices-Cancer (IHC-C) Programme: Pilot randomised trial outcome measurements. (PDF)

## References

[1] Bray F, Laversanne M, Sung H, Ferlay J, Siegel RL, Soerjomataram I, et al. Global cancer statistics 2022: GLOBOCAN estimates of incidence and mortality worldwide for 36 cancers in 185 countries. CA Cancer J Clin. 2024;74(3):229–63. doi: 10.3322/caac.21834 38572751

[2] Sievers Y, Roser K, Scheinemann K, Michel G, Ilic A. The information needs of relatives of childhood cancer patients and survivors: a systematic review of quantitative evidence. Patient Educ Couns. 2024;126:108316. doi: 10.1016/j.pec.2024.108316 38788309

[3] Fletcher C, Flight I, Chapman J, Fennell K, Wilson C. The information needs of adult cancer survivors across the cancer continuum: a scoping review. Patient Educ Couns. 2017;100(3):383–410. doi: 10.1016/j.pec.2016.10.008 27765377

[4] Yamaji N, Nagamatsu Y, Kobayashi K, Hasegawa D, Yuza Y, Ota E. Information needs of children with leukemia and their parents’ perspectives of their information needs: a qualitative study. BMC Pediatr. 2022;22(1):414. doi: 10.1186/s12887-022-03478-w 35831839 PMC9277964

[5] Wieldraaijer T, Duineveld LAM, Bemelman WA, van Weert HCPM, Wind J. Information needs and information seeking behaviour of patients during follow-up of colorectal cancer in the Netherlands. J Cancer Surviv. 2019;13(4):603–10. doi: 10.1007/s11764-019-00779-5 31286386 PMC6677678

[6] Lin L, Koh WL, Huang Q, Lee JK. Breast cancer information behaviours and needs among Singapore women: a qualitative study. Asian Pac J Cancer Prev. 2021;22(6):1767–74. doi: 10.31557/APJCP.2021.22.6.1767 34181332 PMC8418835

[7] Xu AJ, Myrie A, Taylor JI, Matulewicz R, Gao T, Pérez-Rosas V, et al. Instagram and prostate cancer: using validated instruments to assess the quality of information on social media. Prostate Cancer Prostatic Dis. 2021;25(4):791–3. doi: 10.1038/s41391-021-00473-7 34853412

[8] Afful-Dadzie E, Afful-Dadzie A, Egala SB. Social media in health communication: a literature review of information quality. Health Inf Manag. 2023;52(1):3–17. doi: 10.1177/1833358321992683 33818176

[9] Suarez-Lledo V, Alvarez-Galvez J. Prevalence of health misinformation on social media: systematic review. J Med Internet Res. 2021;23(1):e17187. doi: 10.2196/17187 PMC7857950 33470931

[10] Bahar-Ozdemir Y, Ozsoy-Unubol T, Akyuz G. Is YouTube a high-quality source of information on cancer rehabilitation?. J Cancer Surviv. 2022;16(5):1016–22. doi: 10.1007/s11764-021-01093-9 34347245

[11] Warner EL, Kirchhoff AC, Ellington L, Waters AR, Sun Y, Wilson A, et al. Young adult cancer caregivers’ use of social media for social support. Psychooncology. 2020;29(7):1185–92. doi: 10.1002/pon.5402 32364665

[12] Loeb S, Langford AT, Bragg MA, Sherman R, Chan JM. Cancer misinformation on social media. CA Cancer J Clin. 2024;74(5):453–64. doi: 10.3322/caac.21857 38896503 PMC11648589

[13] Johnson SB, Parsons M, Dorff T, Moran MS, Ward JH, Cohen SA, et al. Cancer misinformation and harmful information on Facebook and other social media: a brief report. J Natl Cancer Inst. 2022;114(7):1036–9. doi: 10.1093/jnci/djab141 34291289 PMC9275772

[14] RESIST 2. 2021.

[15] Infodemic. 2020 [cited 4 July 2024]. Available from: https://www.who.int/health-topics/infodemic

[16] health-misinformation-toolkit-english.pdf. Available from: https://www.hhs.gov/sites/default/files/health-misinformation-toolkit-english.pdf

[17] Baguley BJ, Benna-Doyle S, Drake S, Curtis A, Stewart J, Loeliger J. Access to nutrition services and information after active cancer treatment: a mixed methods study. J Cancer Surviv. 2024;18(1):176–85. doi: 10.1007/s11764-023-01352-x 36823493 PMC10866769

[18] ASCO Cancer Opinions Survey. 2018 [cited 2 July 2024]. Available: https://scholar.google.com/scholar_lookup?title=ASCO+2019+Cancer+Opinions+Survey+2019&

[19] Teplinsky E, Ponce SB, Drake EK, Garcia AM, Loeb S, van Londen GJ, et al. Online medical misinformation in cancer: distinguishing fact from fiction. JCO Oncol Pract. 2022;18(8):584–9. doi: 10.1200/OP.21.00764 35357887 PMC9377685

[20] Morgan G, Tagliamento M, Lambertini M, Devnani B, Westphalen B, Dienstmann R, et al. Impact of COVID-19 on social media as perceived by the oncology community: results from a survey in collaboration with the European Society for Medical Oncology (ESMO) and the OncoAlert Network. ESMO Open. 2021;6(2):100104. doi: 10.1016/j.esmoop.2021.100104 33838532 PMC8038939

[21] Bylund CL, Mullis MD, Alpert J, Markham MJ, Onega T, Fisher CL, et al. Clinician communication with patients about cancer misinformation: a qualitative study. JCO Oncol Pract. 2023;19(3):e389–96. doi: 10.1200/OP.22.00526 36626708

[22] Naganathan G, Bilgen I, Cleland J, Reel E, Cil T. #COVID19 and #Breastcancer: a qualitative analysis of tweets. Curr Oncol. 2022;29(11):8483–500. doi: 10.3390/curroncol29110669 36354729 PMC9689212

[23] Leveridge MJ. The state and potential of social media in bladder cancer. World J Urol. 2016;34(1):57–62. doi: 10.1007/s00345-015-1725-y 26590917

[24] Bounegru L, Gray J, Venturini T, Mauri M. A field guide to “Fake News” and other information disorders. SSRN Journal. 2018. doi: 10.2139/ssrn.3097666

[25] Lewandowsky S, Cook J, Ecker U, Albarracín D, Kendeou P, Newman E, et al. The Debunking Handbook 2020. Copyright, Fair Use, Scholarly Communication, etc. 2020. Available from: https://digitalcommons.unl.edu/scholcom/245

[26] Chen X, Hay JL, Waters EA, Kiviniemi MT, Biddle C, Schofield E, et al. Health literacy and use and trust in health information. J Health Commun. 2018;23(8):724–34. doi: 10.1080/10810730.2018.1511658 30160641 PMC6295319

[27] Beaudoin DE, Longo N, Logan RA, Jones JP, Mitchell JA. Using information prescriptions to refer patients with metabolic conditions to the Genetics Home Reference website. J Med Libr Assoc. 2011;99(1):70–6. doi: 10.3163/1536-5050.99.1.012 21243058 PMC3016649

[28] Hopkinson JB. Educational needs of self-care in cachectic cancer patients and caregivers. Curr Opin Oncol. 2023;35(4):254–60. doi: 10.1097/CCO.0000000000000948 37222192

[29] Oxman AD, Chalmers I, Dahlgren A. Key concepts for assessing claims about treatment effects and making well-informed treatment choices (Version 2022). Zenodo. 2022. doi: 10.5281/zenodo.6611932 PMC6290969 30631443

[30] IHC homepage. Informed Health Choices homepage. In: Informed Health Choices [Internet]. 2025 [cited 2 July 2024]. Available from: https://www.informedhealthchoices.org/

[31] Li M, Devane D, Beecher C, Duffy AG, Duggan C, Dowling M, et al. Prioritising Informed Health Choices Key Concepts for those impacted by cancer: a protocol. HRB Open Res. 2022;5:55. doi: 10.12688/hrbopenres.13593.1 37753169 PMC10518847

[32] Li M, Devane D, Beecher C, Dowling M, Duffy AG, Duggan C, et al. Prioritising key concepts for informed health choices in cancer: An evidence-based online educational programme. PEC Innov. 2024;5:100311. doi: 10.1016/j.pecinn.2024.100311 39027229 PMC11254741

[33] Chan A-W, Tetzlaff JM, Gøtzsche PC, Altman DG, Mann H, Berlin JA, et al. SPIRIT 2013 explanation and elaboration: guidance for protocols of clinical trials. BMJ. 2013;346:e7586. doi: 10.1136/bmj.e7586 23303884 PMC3541470

[34] Eysenbach G. CONSORT-EHEALTH: improving and standardizing evaluation reports of Web-based and mobile health interventions. J Med Internet Res. 2011;13(4):e126. doi: 10.2196/jmir.1923 22209829 PMC3278112

[35] Eldridge SM, Chan CL, Campbell MJ, Bond CM, Hopewell S, Thabane L, et al. CONSORT 2010 statement: extension to randomised pilot and feasibility trials. Pilot Feasibility Stud. 2016;2:64. doi: 10.1186/s40814-016-0105-8 27965879 PMC5154046

[36] Schulz KF, Altman DG, Moher D, CONSORT Group. CONSORT 2010 Statement: updated guidelines for reporting parallel group randomised trials. BMC Med. 2010;8:18. doi: 10.1186/1741-7015-8-18 20334633 PMC2860339

[37] Mihuta ME, Green HJ, Shum DHK. Efficacy of a web-based cognitive rehabilitation intervention for adult cancer survivors: a pilot study. Eur J Cancer Care (Engl). 2018;27(2):e12805. doi: 10.1111/ecc.12805 29314350

[38] Dodds SE, Pace TWW, Bell ML, Fiero M, Negi LT, Raison CL, et al. Feasibility of Cognitively-Based Compassion Training (CBCT) for breast cancer survivors: a randomized, wait list controlled pilot study. Support Care Cancer. 2015;23(12):3599–608. doi: 10.1007/s00520-015-2888-1 26275769

[39] Wells-Di Gregorio SM, Marks DR, DeCola J, Peng J, Probst D, Zaleta A, et al. Pilot randomized controlled trial of a symptom cluster intervention in advanced cancer. Psychooncology. 2019;28(1):76–84. doi: 10.1002/pon.4912 30335211

[40] Børøsund E, Varsi C, Clark MM, Ehlers SL, Andrykowski MA, Sleveland HRS, et al. Pilot testing an app-based stress management intervention for cancer survivors. Transl Behav Med. 2020;10(3):770–80. doi: 10.1093/tbm/ibz062 31330023 PMC7413188

[41] Sim J, Lewis M. The size of a pilot study for a clinical trial should be calculated in relation to considerations of precision and efficiency. J Clin Epidemiol. 2012;65(3):301–8. doi: 10.1016/j.jclinepi.2011.07.011 22169081

[42] Lancaster GA, Dodd S, Williamson PR. Design and analysis of pilot studies: recommendations for good practice. J Eval Clin Pract. 2004;10(2):307–12. doi: 10.1111/j.2002.384.doc.x 15189396

[43] Julious SA. Sample size of 12 per group rule of thumb for a pilot study. Pharmaceutical Statistics. 2005;4(4):287–91. doi: 10.1002/pst.185

[44] Whitehead AL, Julious SA, Cooper CL, Campbell MJ. Estimating the sample size for a pilot randomised trial to minimise the overall trial sample size for the external pilot and main trial for a continuous outcome variable. Stat Methods Med Res. 2016;25(3):1057–73. doi: 10.1177/0962280215588241 26092476 PMC4876429

[45] Hertzog MA. Considerations in determining sample size for pilot studies. Res Nurs Health. 2008;31(2):180–91. doi: 10.1002/nur.20247 18183564

[46] QuestionPro. 2022 [cited 7 May 2025]. Available from: https://www.questionpro.com/help/edit-survey.html

[47] Norman CD, Skinner HA. eHEALS: The eHealth Literacy Scale. J Med Internet Res. 2006;8(4):e27. doi: 10.2196/jmir.8.4.e27 17213046 PMC1794004

[48] Chen B, Yi GY, Cook RJ. Weighted generalized estimating functions for longitudinal response and covariate data that are missing at random. J Am Stat Assoc. 2010;105(489):336–53. doi: 10.1198/jasa.2010.tm08551

[49] GDPR - University of Galway. [cited 25 Sept 2024]. Available from: https://www.universityofgalway.ie/data-protection/gdpr/

[50] Joyce S, Shand F, Tighe J, Laurent SJ, Bryant RA, Harvey SB. Road to resilience: a systematic review and meta-analysis of resilience training programmes and interventions. BMJ Open. 2018;8(6):e017858. doi: 10.1136/bmjopen-2017-017858 PMC6009510 29903782

