# Supplementary material for: Evaluating the feasibility and acceptability of the Informed Health Choices-Cancer programme: A pilot randomised trial protocol
Source: PLoS One. 2025 Oct 9;20(10):e0333871. doi: 10.1371/journal.pone.0333871 (PMC12510521; doi:10.1371/journal.pone.0333871)
Supplement: S2 File — (PDF) [file pone.0333871.s002.pdf]

Informed Health Choices-Cancer (IHC-C) Programme

Pilot randomised trial outcome measurements

Please provide your email address *(Please use the same one you gave us earlier)*.

____

Section 1

This section helps us understand how the IHC-C programme worked for you, and what you think about it overall.

(1 Feasibility)

(Technical)

1. How would you rate your ease in accessing the programme?
   - Very difficult
   - Difficult
   - Neutral
   - Easy
   - Very easy

2. Did you experience any technical issues while using the online platform (e.g., difficulties with logging in, accessing materials, or using/navigating the site)?
   - No
   - Yes, minor issues (did not affect my taking part)
   - Yes, significant issues (affected my taking part)
   - Other (please specify)

(2 Acceptability)

(Participant burden)

1. How much effort did you put into engaging with the programme? This includes things like understanding what you needed to learn or doing the activities.
   Very low effort
   Low effort
   Moderate effort
   High effort
   Very high effort

2. Was the amount of work/effort required for the programme okay/acceptable?
   - Yes, the effort was acceptable
   - No, the effort was too burdensome

(General acceptability – Content)

3. How would you rate the content of the programme?
   Very unacceptable
   Unacceptable
   Neither acceptable nor unacceptable
   Acceptable
   Very acceptable

4. I found the programme easy to understand.
   Strongly disagree

Disagree
Neither agree nor disagree
Agree
Strongly agree

5. I found the programme relevant to cancer.
   Strongly disagree
   Disagree
   Neither agree nor disagree
   Agree
   Strongly agree

6. I found what I learnt from this programme useful in my daily life.
   Strongly disagree
   Disagree
   Neither agree nor disagree
   Agree
   Strongly agree

(General acceptability – Format)

7. How would you rate the acceptability of the format (e.g., use of videos, images, or articles) of the programme?
   Very unacceptable
   Unacceptable
   Neither acceptable nor unacceptable
   Acceptable
   Very acceptable

8. I found the use of videos, text, articles, and case stories, etc effective.
   Strongly disagree
   Disagree
   Neither agree nor disagree
   Agree
   Strongly agree

9. I found these formats easy to apply and follow.
   Strongly disagree
   Disagree
   Neither agree nor disagree
   Agree
   Strongly agree

(General acceptability – Duration)

10. Overall, how did you feel about how long the programme lasted?
    Very unacceptable
    Unacceptable
    Neither acceptable nor unacceptable
    Acceptable
    Very acceptable

11. I thought each lesson was just the right size/length to finish.

Strongly disagree
Disagree
Neither agree nor disagree
Agree
Strongly agree

(General acceptability)
12. How acceptable did you find the programme overall?
Very unacceptable
Unacceptable
Neither acceptable nor unacceptable
Acceptable
Very acceptable

13. I found the programme met my expectations.
Strongly disagree
Disagree
Neither agree nor disagree
Agree
Strongly agree

(*General acceptability – Willingness to recommend the programme)*
14. I would recommend this programme to others who are impacted by cancer.
Strongly disagree
Disagree
Neither agree nor disagree
Agree
Strongly agree

(3 Secondary outcomes (question source: review summary and team development)
3.1 Cognition and behaviour change)
1. My understanding of cancer-related information and misinformation has improved after taking part in this programme.
- Strongly disagree
- Disagree
- Neither agree nor disagree
- Agree
- Strongly agree

2. I have changed (or plan to change) how I deal with cancer information, like checking where it comes from before liking, sharing, or commenting.
- Strongly disagree
- Disagree
- Neither agree nor disagree
- Agree
- Strongly agree

Section 2
This section is about how you use the internet to look for information. There are 10 short questions.

(3.2 eHealth Literacy Scale)

1. How useful do you feel the Internet is in helping you in making decisions about your health?

    Not useful at all
    Not useful
    Unsure
    Useful
    Very useful

2. How important is it for you to be able to access health resources on the internet?
    - Not important at all
    - Not important
    - Unsure
    - Important
    - Very important

3. I know what health resources are available on the internet
    - Strongly Disagree
    - Disagree
    - Undecided
    - Agree
    - Strongly Agree

4. I know where to find helpful health resources on the internet
    - Strongly Disagree
    - Disagree
    - Undecided
    - Agree
    - Strongly Agree

5. I know how to find helpful health resources on the internet
    - Strongly Disagree
    - Disagree
    - Undecided
    - Agree
    - Strongly Agree

6. I know how to use the internet to answer my questions about health
    - Strongly Disagree
    - Disagree
    - Undecided
    - Agree
    - Strongly Agree

7. I know how to use the health information I find on the internet to help me
    - Strongly Disagree
    - Disagree
    - Undecided
    - Agree
    - Strongly Agree

8. I have the skills I need to evaluate the health resources I find on the internet

- Strongly Disagree
- Disagree
- Undecided
- Agree
- Strongly Agree

9. I can tell high quality health resources from low quality health resources on the internet

- Strongly Disagree
- Disagree
- Undecided
- Agree
- Strongly Agree

10. I feel confident in using information from the internet to make health decisions

- Strongly Disagree
- Disagree
- Undecided
- Agree
- Strongly Agree
